# Detection of free circulating Epstein-Barr virus DNA in plasma of patients with Hodgkin's disease

**DOI:** 10.1590/S1516-31802006000300009

**Published:** 2006-05-04

**Authors:** Juliane Garcez Musacchio, Maria da Glória da Costa Carvalho, José Carlos Oliveira de Morais, Nathalie Henriques Silva, Adriana Scheliga, Sérgio Romano, Nelson Spector

**Keywords:** DNA, Epstein-Barr virus, Hodgkin disease, Lymphoma, Polymerase chain reaction, DNA, Vírus Epstein-Barr, Doença de Hodgkin, Linfoma, Reação em cadeia da polimerase

## Abstract

**CONTEXT AND OBJECTIVE:**

Free circulating Epstein-Barr virus (EBV) DNA is often present in the plasma of Hodgkin's disease patients. The aim here was to evaluate the prevalence of this finding, its correlation with the immunohistochemical expression of LMP-1 (latent membrane protein 1) and the influence of other clinical factors.

**DESIGN AND SETTING:**

Prospective study in two public tertiary institutions: Hematology Service, Universidade Federal do Rio de Janeiro, and Oncology Service, Instituto Nacional do Câncer, Rio de Janeiro.

**METHODS:**

A cohort of 30 patients with newly diagnosed Hodgkin's disease was studied. The control group consisted of 13 healthy adult volunteers. EBV DNA was determined by conventional polymerase chain reaction (PCR).

**RESULTS:**

The median age was 28 years, and 16 patients were women. Advanced disease was present in 19 patients, and six were HIV-positive. EBV DNA was present in the plasma of 13 patients and one control (43% versus 8%, p = 0.03). EBV DNA prevalence was higher in HIV-positive patients (100% versus 29%, p = 0.0007) and those with advanced disease (63% versus 9%, p = 0.006). Among HIV-negative patients alone, EBV DNA prevalence remained higher in those with advanced disease. EBV DNA was found in 10/11 patients with LMP-1 expression in the lymph nodes, and in 3/19 without LMP-1 expression (kappa coefficient = 0.72).

**CONCLUSION:**

EBV DNA was present in 91% of patients with EBV-associated Hodgkin's disease, and in all patients with HIV-associated Hodgkin's disease. EBV DNA prevalence was higher in patients with advanced disease, irrespective of HIV status.

## RESUMO

**CONTEXTO E OBJETIVO:**

O DNA do vírus Epstein-Barr (EBV) está freqüentemente presente no sangue periférico de pacientes com doença de Hodgkin. O objetivo deste estudo foi avaliar a prevalência deste achado, e correlacioná-lo com a expressão imunoistoquímica da LMP-1 (*latent membrane protein* 1) e a presença de outros fatores clínicos.

**TIPO DE ESTUDO E LOCAL:**

Estudo prospectivo realizado no Serviço de Hematologia da Universidade Federal do Rio de Janeiro e no Serviço de Oncologia do Instituto Nacional do Câncer, Rio de Janeiro, Brasil.

**MÉTODOS:**

Trinta pacientes com doença de Hodgkin recém-diagnosticada foram estudados, assim como um grupo controle composto por 13 indivíduos saudáveis. O DNA do EBV no plasma foi determinado pela reação em cadeia da polimerase (PCR) convencional.

**RESULTADOS:**

A idade mediana foi 28 anos e 16 pacientes eram do sexo feminino. A doença disseminada esteve presente em 19 pacientes e seis eram HIV+. O DNA do EBV foi detectado no plasma de 13 pacientes e um controle (43% *versus* 8%, p = 0,03). A prevalência do DNA do EBV foi maior nos pacientes HIV+ (100% *versus* 29%, p = 0,0007) e naqueles com doença disseminada (63% *versus* 9%, p = 0,006). Quando somente os pacientes HIV-negativos foram analisados, a prevalência do DNA do EBV permaneceu maior nos pacientes com doença disseminada. A prevalência do DNA do EBV variou de acordo com o subtipo histológico: foi de 32% nos pacientes com esclerose nodular e de 100% nos pacientes com celularidade mista e depleção linfocítica (p = 0,02). O DNA do EBV foi encontrado em 10/11 pacientes com a expressão da LMP-1 em linfonodos, e em 3/19 pacientes sem a expressão da LMP-1 (coeficiente de kappa = 0,72).

**CONCLUSÕES:**

O DNA circulante do EBV foi detectado no plasma de 91% dos pacientes com doença de Hodgkin associada ao EBV, e em todos os pacientes com doença de Hodgkin associada ao HIV. A prevalência do DNA circulante do EBV foi maior nos pacientes com doença avançada, independentemente do status para o HIV.

## INTRODUCTION

Epstein-Barr virus (EBV), a lymphotropic herpesvirus that is widespread in the human population, is associated with approximately 40% of Hodgkin's disease cases. Its prevalence is higher in developing countries, where a higher proportion of Hodgkin's disease cases are subclassified as mixed cellularity.^1^

The demonstration that EBV genomes are present in the Hodgkin-Reed Sternberg (H-RS) cells of a proportion of Hodgkin's disease cases provided important new evidence of its role in the pathogenesis of the disease.^2^ In patients with EBV-associated Hodgkin's disease, H-RS cells express high levels of latent membrane protein 1 (LMP-1), which functions as a constitutively activated tumor necrosis factor receptor. Many of the growth transforming effects of LMP-1 derive from its ability to activate various signaling pathways, including nuclear factor κB.^3^

Small amounts of free EBV DNA circulate in the plasma of both healthy individuals and cancer patients.^4^ Some studies have reported the presence of EBV DNA in the peripheral blood of patients with EBV-associated malignancies, such as nasopharyngeal carcinoma, NK/T cell lymphoma and post-transplant lymphoproliferative disease (PTLD),^4-8^ but there is still only limited experience of EBV in Hodgkin's disease. Gallagher et al. reported that the conventional polymerase chain reaction (PCR) detected 90% of known EBV-positive Hodgkin's disease cases.^9^ Quantitative PCR appeared to be less sensitive but more specific. In two other studies, a reduction in plasma viral load to low or undetectable levels was observed in patients who obtained complete remission, whereas in poorly responding patients, disease progression was associated with rapidly increasing DNA levels.^10,11^ Thus, analysis of plasma EBV DNA might be of value for diagnostic and prognostic evaluation and follow-up of Hodgkin's disease patients.

## OBJECTIVE

The aim of this study was to confirm whether free circulating EBV DNA can be consistently detected in patients with EBV-associated Hodgkin's disease, and to verify possible associations with such patients’ clinical characteristics.

## METHODS

### Patients

Between November 2001 and November 2003, plasma samples from 30 consecutive patients with newly diagnosed Hodgkin's disease were collected before their treatment started at the Hematology Service of Universidade Federal do Rio de Janeiro and the Oncology Service of Instituto Nacional do Câncer (two public tertiary institutions). Their diagnoses and histological classifications were confirmed in a pathological review performed by two of the authors together (SR and JCM). The presence of EBV in tumor cells was assessed by the immunohistochemical expression of the LMP-1 protein, using a cocktail of monoclonal antibodies (CS1-4, Dako^®^), as described previously.^12^

Patient evaluation included a complete history, physical examination, complete blood count with differential count, biochemical profile, HIV serology, chest radiography, computed tomography of the chest and abdomen, and bone marrow biopsy. Blood samples were collected for EBV DNA analysis.

Blood samples from 13 healthy volunteers were also analyzed. They were all health workers at our hospital, their median age was 29 years, and seven were women.

The study was approved by the Institutional Review Boards of the two institutions. Informed consent was obtained from all patients.

### DNA extraction from plasma samples

DNA extraction was performed using the QIAamp Blood Kit (Qiagen, Uniscience, Brazil). The steps were performed according to the supplier's protocol and samples were resuspended in 200 μl H_2_O. DNA was stored at – 80^°^ C.

### Conventional polymerase chain reaction

Initially, EBV DNA polymerase chain reaction (PCR) was carried out using primers derived from the BamH1 W repeat sequence of EBV, as previously described.^13^ The PCR routine was performed using 2 μl of the DNA extracted from plasma. PCR reactions were performed with 2.5 units/μl Amplitaq (Perkin-Elmer, Branchburg, New Jersey), 1.5 mM MgCl_2_, 25 mM dNTPs, 10 x buffer and 10 μM of each primer, in a 50 μl reaction mix. A negative water control and a positive control consisting of DNA from the EBV-positive Raji cell line were included in each assay. Thermal cycling was performed using the following conditions: initial denaturation at 95^°^ C for five minutes, followed by 40 cycles of ramping to 94^°^ C over one min; 94^°^ C for 30 sec; cooling to 55^°^ C over two min; 55^°^ C for 10 sec; heating to 72^°^ C over one min; 72^°^ C for 30 sec; followed by a final extension step at 72^°^ C for seven min.^9^ PCR products were analyzed by electrophoresis on 10% polyacrylamide gels. All plasma DNA samples were also subjected to PCR analysis for the p53 gene, which served as a control for the ability to amplify the plasma DNA.

### Statistical analysis

Fisher's exact test (two-sided) was used to assess the association between categorical variables. The kappa coefficient was calculated to estimate the concordance between EBV detection methods. Statistical analysis was performed using the softwares Epi-Info 6.0 and PEPI 4.0.

## RESULTS

The patients’ characteristics, EBV status in tumor cells and pre-treatment plasma EBV DNA are summarized in Tables 1 and 2. Tissue samples were available from all 30 patients for evaluating their EBV status.

**Table 1.** Clinical characteristics and plasma Epstein-Barr virus (EBV) DNA of 30 patients with Hodgkin's disease

Characteristics	n (%)
**Median age**	28
Range	10-57 years
**Sex**	
Female	16 (53)
Male	14 (47)
Stage (Ann Arbor method)*	
Localized	11 (37)
Disseminated	19 (63)
**HIV-positive Status**	6 (20)
**Histopathology subtype†**	
Nodular sclerosis	25 (86)
Mixed cellularity	3 (11)
Lymphocytic depletion	1 (3)
**LMP-1 protein**	
Positivity	11 (37)
**Plasma EBV DNA**	
Detectable	13 (43)

*EBV = Epstein-Barr virus.*

* *Localized disease: stages IA, IB and IIA; disseminated disease: stages IIB, IIIA, IIIB, IVA and IVB;*

† *One patient unclassified; diagnosis from bone marrow specimen.*

**Table 2.** Clinical characteristics and plasma Epstein-Barr virus (EBV) DNA of each patient with Hodgkin's disease

Patients	Age	Sex	Stage	Subtype	IHC	HIV	DNA
1	43	M	II B	NS	-	-	-
2	56	F	IV B	LD	+	-	+
3	15	F	IV B	NS	+	-	+
4	18	F	II A	NS	-	-	-
5	49	F	IV B	*	+	+	+
6	22	M	II A	NS	-	-	-
7	46	M	II A	NS	-	-	-
8	32	M	III A	NS	-	-	-
9	19	F	II A	NS	-	-	-
10	21	M	III A	NS	+	-	+
11	20	F	II A	NS	-	-	+
12	10	F	IV B	NS	+	+	+
13	25	F	IV B	NS	-	-	+
14	23	F	IV B	NS	-	-	+
15	57	M	III B	NS	+	+	+
16	44	M	IV B	MC	+	+	+
17	34	F	I A	NS	-	-	-
18	24	F	III B	NS	-	-	-
19	34	M	II A	NS	-	-	-
20	41	F	IV B	MC	+	+	+
21	31	F	III B	NS	-	-	-
22	27	M	III B	NS	+	-	+
23	29	F	II B	NS	-	-	-
24	28	M	II A	NS	-	-	-
25	29	M	II A	NS	-	-	-
26	15	M	II B	NS	+	-	-
27	46	M	II A	NS	-	-	-
28	27	F	II B	NS	-	-	-
29	44	M	III B	MC	+	+	+
30	25	F	II A	NS	-	-	-

*IHC = imunohistochemistry (for LMP-1); HIV = status of human immunodeficiency virus; F = female; M = male; NS = nodular sclerosis; LD = lymphocytic depletion; MC = mixed cellularity;*

* *diagnosis in bone marrow specimen.*

Prior to treatment, plasma EBV DNA was detected in 13 of the 30 patients (43%). In contrast, plasma EBV DNA was detected in only one of the 13 control subjects (p = 0.03; 95% CI for the difference between proportions = 0.12-0.58).

**Figure 1:**
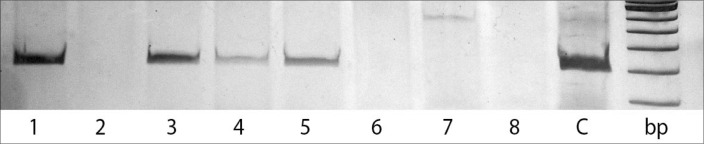
Detection of Epstein-Barr virus (EBV) DNA by polymerase chain reaction (PCR); electrophoresis on 10% polyacrylamide gels in buffer TBE 1x; bp: DNA size marker; C: positive control, DNA from Raji cell line. Samples were from: EBV-associated Hodgkin's Disease, lanes 1, 3, 4 and 5; non-EBV-associated Hodgkin's Disease, lanes 2 and 7; healthy individuals, lanes 6 and 8.

Circulating EBV DNA was found in 10 of the 11 LMP-1 positive patients, and in only three of the 19 patients without LMP-1 expression in the lymph nodes (kappa coefficient = 0.72). Figure 1 shows the presence of EBV DNA in the serum of four patients.

According to the Ann Arbor staging method, advanced disease (defined as stages IIB-IV) was present in 19 patients and the frequency of EBV DNA was higher in this group (63% versus 9%, p = 0.006, 95% CI = 0.26-0.81). Plasma EBV DNA was found in all HIV-positive patients (100% versus 29%, p = 0.0007). When only the 24 HIV-negative patients were analyzed, the frequency of EBV DNA remained higher in the 13 patients with advanced disease (54% versus 9%, p = 0.03)

## DISCUSSION

The presence of tumor-derived DNA in the plasma and serum of cancer patients opens up new possibilities for detecting and monitoring cancer.

In the present study, 43% of the patients with Hodgkin's disease had EBV DNA that was detectable by conventional PCR in the plasma prior to treatment, whereas only one healthy individual (8%) was positive for plasma EBV DNA (p = 0.03). Moreover, plasma EBV DNA was present in almost every patient (10/11, 91%) with proven LMP-1 in the lymph nodes, and also in 3/19 patients (16%) without LMP-1. Whether the latter were false-positive results from the PCR or false-negative results from the LMP-1 staining method remains to be elucidated. In the present study, EBV encoded RNA (EBER) *in situ* hybridization was not performed, but the correlation with LMP-1 was usually good.^14^

Gallagher et al.^9^ observed similar results from patients selected retrospectively on the basis of their known EBV status: 91% of their patients with EBV-associated disease were positive by conventional PCR, while 26% of patients with non-EBV-associated Hodgkin's disease were positive. Subsequently, a subgroup of their patients was also studied by quantitative PCR. With this method, only 69% of the patients with EBV-associated disease and 10% of the patients with nonEBV associated Hodgkin's disease were positive. The authors speculated that this lower positivity might have been due to technical reasons, such as the smaller amount of DNA used in RT-PCR. However, as expected, the median amount of EBV DNA detected by RT-PCR was much higher in patients with EBV-associated Hodgkin's disease.

The good correlation between the EBV status of the involved lymph nodes examined and the ability to detect EBV DNA in plasma suggests that H-RS cells are the most likely source of viral DNA. Support for this idea is provided by Kornacker et al.,^15^ who demonstrated identical immunoglobulin gene rearrangements in biopsy and blood samples from a patient with Hodgkin's disease.

A strong correlation between plasma EBV DNA and HIV status was observed. Virtually all cases of Hodgkin's disease in HIV-infected patients are EBV-associated,^16,17^ and in our series the prevalence of free EBV DNA was 100%. Free circulating EBV DNA could have clinical use in patients with HIV-related Hodgkin's disease, both as an indicator of complete remission and as a tool for following up patients who are in complete remission.^18^

A strong correlation was also observed between the presence of free circulating EBV DNA and the presence of advanced Hodgkin's disease. A similar correlation has been observed in patients with nasopharyngeal carcinoma: plasma cell-free EBV DNA levels in advanced cases were significantly higher than those in early-stage nasopharyngeal cases.^19^ These results again suggest that free EBV DNA is related to the tumor mass, and that its analysis might be useful as a molecular marker for the detection and follow-up of EBV-related diseases.

The control group had the same median age and sex distribution as the patients with Hodgkin's disease. The clinical significance of the presence of plasma EBV DNA in one healthy control is unclear. Such positive findings in control groups have been observed before in developing countries, with a prevalence of 7%-13%.^19,20^ However, in developed countries, EBV DNA has never been detected in the plasma or serum of healthy, non-immunosuppressed children or adults.^21^ Therefore, this discrepancy might be related to yet undefined socioeconomic conditions.

In studies using large DNA banks, Hodgkin's disease has been shown to develop as late as seven years after free EBV DNA was detected.^22^ For this reason, the only control volunteer with a positive result in our study is being monitored for clinical signs of EBV-associated diseases.

It is of interest that, in one study that detected plasma EBV DNA in controls, the levels of plasma EBV DNA in patients with nasopharyngeal carcinoma were much higher than those in controls with detectable plasma EBV DNA.^6^ Thus, quantitative plasma EBV DNA might prove more useful as a screening method for EBV-associated diseases in high-prevalence areas.

## CONCLUSIONS

In summary, free circulating EBV DNA was frequently found in Hodgkin's disease patients before treatment by conventional PCR, and its prevalence was especially high in patients with advanced-stage disease and HIV-related Hodgkin's disease. Further longitudinal studies are clearly needed in order to evaluate the diagnostic impact of this finding on the monitoring of treatment response and remission status among Hodgkin's disease patients. The use of quantitative PCR in a larger population is likely to shed light on many of the questions raised by this study.
